# Supplementary material for: A Critical View of the Application of the APEX Software (Aqueous Photochemistry of Environmentally-Occurring Xenobiotics) to Predict Photoreaction Kinetics in Surface Freshwaters
Source: Molecules. 2019 Dec 18;25(1):9. doi: 10.3390/molecules25010009 (PMC7017383; doi:10.3390/molecules25010009)
Supplement: Supplementary file 1 [file molecules-25-00009-s001.zip › 3.2.4_gcc-4.4.0.zip!/3.2.4_gcc-4.4.0/3.2.4_gcc-4.4.0/doc/octave/html/interpreter/Data-Containers.html]

Data Containers - Untitled


Next: Variables,
Previous: Strings,
Up: Top


---

## 6 Data Containers

Octave includes support for two different mechanisms to contain
arbitrary data types in the same variable. Structures, which are C-like,
and are indexed with named fields, and cell arrays, where each element
of the array can have a different data type and or shape. Multiple
input arguments and return values of functions are organized as
another data container, the comma separated list.

- Data Structures- Cell Arrays- Comma Separated Lists
